# Cross-Sectional Associations of 24-Hour Sedentary Time, Physical Activity, and Sleep Duration Compositions with Sleep Quality and Habits in Preschoolers

**DOI:** 10.3390/ijerph17197148

**Published:** 2020-09-29

**Authors:** Christine W. St. Laurent, Sarah Burkart, Katrina Rodheim, Robert Marcotte, Rebecca M. C. Spencer

**Affiliations:** 1 Department of Psychological and Brain Sciences, University of Massachusetts, Amherst, MA 01003, USA; Katrina.Rodheim@colorado.edu; 2 Department of Exercise Science, University of South Carolina, Columbia, SC 29208, USA; sburkart@mailbox.sc.edu; 3 Department of Kinesiology, University of Massachusetts, Amherst, MA 01003, USA; rmarcotte@umass.edu

**Keywords:** time-use, 24-h activity cycle, children, sleep, physical activity, sedentary behavior

## Abstract

Although some studies indicate physical activity and sleep quality are positively associated in children, most reports examined physical activity independent of other 24-h behaviors and focused on older children. The aim of this cross-sectional study was to examine the predicted changes in sleep efficiency and habits when reallocating time between movement behaviors using compositional isotemporal substitution in preschool-aged children. Accelerometers were worn by 288 participants (51.6 ± 9.5 months) for up to 16 days. Sleep outcomes included sleep efficiency, nap frequency, sleep disturbances, and bedtime resistance. Compositional isotemporal substitution analyses demonstrated that the combined effect of 24-h movement behaviors was associated with sleep efficiency (*p* < 0.001) and nap frequency (*p* < 0.003). When sleep increased by 30 min at the expense of stationary time or light physical activity, estimates of sleep efficiency and bedtime resistance decreased while nap frequency increased. When stationary time increased by 30 min from moderate to vigorous physical activity, estimated sleep efficiency increased and sleep disturbances decreased. Although this study presents preliminary evidence that 24-h movement behavior compositions in early childhood are associated with sleep quality and nap frequency, estimated effects from theoretical time reallocations across sleep outcomes were mixed.

## 1. Introduction

Physical activity and sleep are both important modifiable behaviors that contribute to physical and cognitive health in young children [1,2,3]. Together, sedentary behaviors, physical activity (i.e., light and moderate-to-vigorous physical activity) and sleep have been coined 24-h movement behaviors and some national and international health organizations have begun to promote comprehensive movement behavior guidelines for a 24-h cycle [4,5]. For example, the World Health Organization (WHO) has recently published guidelines with sleep, sedentary behavior, and physical activity recommendations for children under 5 years of age [6]. The WHO guidelines recommend that preschool children (i.e., ages 3 to 4 years) participate in at least 180 min of physical activity, engage in no more than 60 min of sedentary screen time, and obtain 10 to 13 h of good quality sleep in a 24-h day. However, some nations such as the United States have advised that additional evidence is needed to develop such guidelines, particularly for early childhood [7]. Thus, there is a need to examine the interactive nature of these 24-h movement behaviors in young children to not only understand the influence these behaviors have on various health outcomes, but also how they influence one another. 

While it appears that the time spent in physical activity (particularly moderate-to-vigorous physical activity (MVPA)) is positively associated with sleep outcomes in adults, the evidence is less consistent in children [7]. In a recent review that synthesized the reported relationships between physical activity and sleep in children and adolescents, among 100 observational studies, only 17 included participants under the age of 6 years [8]. Among these, most studies focused on sleep duration or time in bed as the primary measure of sleep [9,10,11,12,13,14,15,16,17,18,19,20]. Most studies have focused primarily on overnight sleep, ignoring napping which is prevalent in early childhood.

In addition to sleep duration, other measures of sleep quality such as sleep efficiency (the percentage of time that an individual is actually asleep between sleep onset and wake onset) and sleep habits (e.g., sleep disturbances, behaviors, and routines) are important indicators of sleep health [21,22]. Sleep disturbances are evident in children as young as 3 to 6 years of age and appear to persist into middle childhood [23]. The few studies that have assessed the relation between physical activity and sleep efficiency and habits in early life have been limited to infants and toddlers [24,25,26,27] or used proxy measures of physical activity (i.e., sports participation) [28]. Importantly, these studies are also limited in that measures of physical activity have been studied in isolation, or statistical models have only partially adjusted for time spent in other 24-h movement behaviors. 

Given that measures of physical activity and sleep duration are co-dependent and occur within a finite period of time (i.e., 24 h), use of traditional analyses that do not account for multicollinearity of these behaviors may lead to inaccurate interpretations [29,30]. Researchers have begun to incorporate compositional data analysis (CoDA) to explore the compositions of 24-h movement behaviors on various health outcomes [31,32,33,34,35]. A few recent research groups have used such an approach to examine relationships between 24-h movement behaviors and various outcomes of young children [36,37,38]. As highlighted by the recently developed Framework for Viable Integrative Research in Time-Use Epidemiology (VIRTUE) [39], further studies are needed that use objective measures and appropriate analyses of 24-h movement behaviors to examine additional health outcomes. The VIRTUE framework also recommends that estimating effects of time reallocation on a particular health outcome using compositional isotemporal substitution is a meaningful way to interpret results from compositional analyses. 

Examining the potential interacting effects of daytime and 24-h movement behaviors on various sleep related outcomes while accounting for the compositional nature of these measures may better inform movement guidelines for young children. Therefore, the objective of this study was to examine the predicted changes in sleep efficiency and habits when reallocating time between movement behaviors using compositional isotemporal substitution in preschool-aged children.

## 2. Materials and Methods 

This study employed cross-sectional analyses using data from a larger randomized controlled trial examining the benefits of napping on memory in preschool-aged children. Data were collected between 2013 and 2019. The Strengthening the Reporting of Observational Studies in Epidemiology (STROBE; 2014) guidelines were followed in the reporting of this study [40]. Written informed consent and parent permission were completed by parents or guardians and child participants gave their verbal assent for inclusion before they participated in the study. The study was conducted in accordance with the Declaration of Helsinki, and the protocol was approved by the University of Massachusetts Amherst Institutional Review Board (approved 15 December 2011; protocol ID: 2011-1152).

### 2.1. Participants

**Figure 1:**
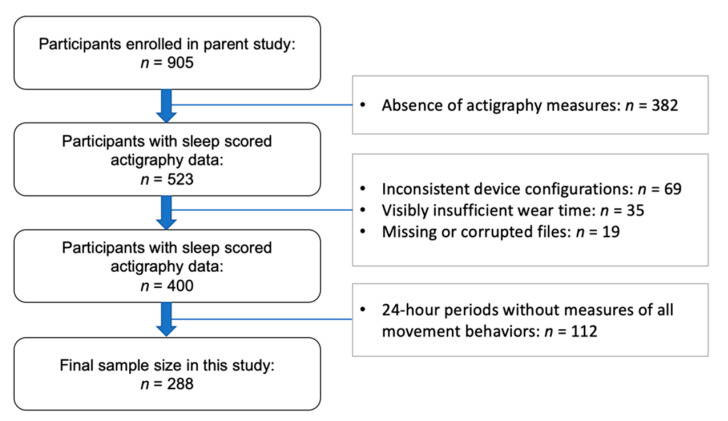
STROBE study flow chart of participants.

Participants consisted of preschool students attending childcare centers in Western Massachusetts. Eligibility criteria included children aged 33 to 71 months (the typical age range for preschool in the United States), normal or corrected-to-normal vision and hearing, absence of a current or past diagnosis of sleep disorder or developmental disability, no current use of sleep-effecting or psychotropic medications, and no travel outside of the local time zone within one week prior to the study. Participant data was excluded from the present analysis if they did not meet minimum wear time compliance (Figure 1).

### 2.2. Measures 

#### 2.2.1. Exposure Measures

Twenty-four-hour movement behavior data was measured via actigraphy for up to 16 days. Actigraphy was collected with the Actiwatch Spectrum (Philips Respironics, Bend, OR, USA), a wrist-worn, water-resistant triaxial accelerometer that has off-wrist detection. The devices were configured to collect data in 15-s epochs, and participants were encouraged to press a button on the device (an event marker) any time that they laid down to go to sleep or when they woke up from sleep. Actigraphy data was downloaded with Actiware software and were then scored for sleep or wake using 15-s epochs. Epochs were designated as sleep, wake, or excluded (i.e., if it was designated as off-wrist).

Sleep diaries were used to aid in actigraphy scoring. Parents and guardians were asked to report on nap and overnight sleep periods at home by recording the time the child was in bed, time it took the child to fall asleep, wake time, and any periods that the watch was taken off. Preschool classroom teachers also kept a nap diary for school days and recorded if children napped and if so, the time nap started and ended. Intervals were designated as sleep episodes using a combination of sleep diaries and event markers. If sleep diary entries or event markers were unavailable (i.e., in 21.5% of participants), then the first 3 consecutive minutes of sleep were used to define sleep onset and the last 5 consecutive minutes of sleep defined wake onset for sleep episodes. The program’s default algorithm was applied to both daytime (i.e., nap) and nighttime sleep episodes [41]. Data were cleaned and collapsed in SPSS (Version 25.0, IBM Corp., Armonk, NY, USA). Sleep variables in the present analyses included nap time in bed (min), nighttime time in bed (min), and total 24-h (i.e., nap and nighttime) time in bed (min).

Due to the protocol of the parent study, one afternoon (i.e., noon to 4 pm) per week was considered experimental (children were either wake- or nap-promoted), and therefore, those intervals were excluded from actigraphy analyses. Using cut points proposed by Ekblom et al. [42] (i.e., sedentary ≤ 79 counts, light intensity physical activity = 80 to 261 counts, moderate-to-vigorous intensity physical activity ≥ 262 counts) wake epochs were categorized into time spent (minutes) in stationary time or physical activity intensities. These cut points were validated in our lab against waist worn ActiGraph accelerometers and direct observation in preschoolers [43]. Given that estimating sedentary behavior should involve the assessment of posture, and this component was not measured with the Actiwatch Spectrum accelerometers, we refer to our measure of sedentary behavior as stationary time [44].

#### 2.2.2. Outcome Measures 

Sleep quality and behavior measures were derived from actigraphy and a parent-reported questionnaire. Sleep efficiency served as an indicator of sleep quality and was determined via actigraphy and calculated as the actual time asleep divided by time in bed. Nap habituality was also derived through actigraphy. Weekly nap frequency (i.e., naps per week) was calculated using the number of naps taken over days that a nap opportunity was present (i.e., the monitor was worn, and afternoon intervals were not excluded). As a subjective measure of sleep behaviors and habits, the Child Sleep Habits Questionnaire (CSHQ) was completed by parents/guardians. The CSHQ is a reliable questionnaire to measure sleep problems in both community (Cronbach’s alpha = 0.68) and clinical (Cronbach’s alpha = 0.78) samples of children ages 4 to 10 years [21] and has been used previously in preschool children [45]. In the CSHQ, a series of statements (45 items) regarding sleep-related behaviors and practices are presented and responders are asked to select one of three responses that are true for their child over the previous week (i.e., “usually: 5–7 days/week”, “sometimes: 2–4 days/week, “rarely: 0–1 days/week”). Scoring for each item was on a 3-point scale (with some items reversed coded), with a higher score indicating more disturbed sleep. In this study, of interest was Total Sleep Disturbances subscale score (maximum possible score = 99), as well as the Bedtime Resistance subscale score (maximum possible score = 15). 

#### 2.2.3. Covariate Measures

Parents and guardians completed a questionnaire to report demographic information about their family and preschool child. Variables included child’s age, sex, and socioeconomic status (SES). An SES score (range = 0 to 7) was calculated from the parents’ highest level of education, household income, and employment status and then indexed as low (0 to 2), middle (3 to 4), or high (5 to 7) [46]. For body mass index (BMI), researchers measured height and weight in the preschool classroom. Using date of birth and date of measurement, BMI score and percentile were calculated using the Centers for Disease Control’s calculations for children [47].

### 2.3. Analysis 

Descriptive statistics were calculated in Stata (Version 16., StataCorp LLC, College Station, TX, USA). Compositional data analyses were conducted in R (http://cran.r.porject.org) using the packages Compositions [48] and robCompositions [49]. Statistical significance was set a priori at *p* < 0.05.

Only 24-h days that contained sufficient wake wear time (i.e., a minimum of 480 min) and a full night of actigraphy data for that subsequent night were included in analyses. Although a minimum of 600 min of wear time is generally recommended for accelerometry measurement [50], given that many of our participants napped for 60 to 120 min during the day, 480 min of wear time during wake was selected for this study. Similar to a recent report by Taylor et al. [38] to keep overnight sleep bouts within the same block, each 24-h “day” started with wake onset of a given day and concluded with wake onset the following day. Given that each day may not add up exactly to 1440 min, data were normalized by dividing the time spent in each movement behavior by the total wear time of that block (i.e., wake wear time plus total time in bed). Given the high compliance on weekdays, participants without any valid weekend days were still included. However, we conducted independent *t* tests to assess if sleep outcomes varied by participants with weekend days versus participants without any valid weekend days.

Descriptive statistics were calculated for all variables. Arithmetic means and standard deviations were determined for standard continuous data, and percentages were calculated for standard categorical data. For compositional data, geometric means for each behavior component were divided by the sum of the component geometric means. Each component was then multiplied by 1440 min to normalize the data to 24 h. A variance matrix was calculated for the four behavior components of the 24-h composition to illustrate dispersion of the compositional data (i.e., the log of all pair-wise ratios between behavior components). Lower levels of co-dependence between components are indicated by higher values and higher levels of co-dependence are indicated by values closer to zero.

To examine the general associations between the 24-h movement behaviors and sleep outcomes, compositional data was expressed as isometric log-ratio (ilr) coordinates to be entered into models as described by previous CoDA methods papers [31,32]. Similar to recent compositional isotemporal substitution reports, sequential binary partition was used to determine the ilr coordinates, and the selection was arbitrary given that the results for the compositional time reallocations will not vary with the use of a different partitioning system [51,52]. For the 24-h behavior composition (i.e., four components), this resulted in three ilr-transformed coordinates. Linear regression models incorporated ilr-coordinates of the compositional data and covariates (i.e., age, gender, and BMI percentile). Models were checked for linear regression assumptions (i.e., linearity, normality, homoscedasticity, and presence of outlying data). The car: Anova() function was used to test the significance of the explanatory variables by sequentially testing each covariate of the models.

To determine the associations of time reallocations of 24-h movement behaviors and sleep outcomes, the compositional isotemporal substitution method described by Dumuid et al. [51] and utilized by recent CoDA articles [38,52,53] was followed. First, predicted estimates of sleep outcomes (i.e., sleep efficiency, Total Sleep Disturbances score, Bedtime Resistance Score, and nap frequency) were calculated from the linear models of the “baseline” composition. Next, linear models analyzed new compositions that accounted for a 30-min reallocation from one behavior to another while keeping the other two behaviors constant to predict “new” estimates of sleep outcomes. For each pair-wise combination of time reallocation, the difference in predictions was calculated.

## 3. Results

### 3.1. Descriptive Statistics

The study characteristics are presented in Table 1. A total of 288 participants had sufficient actigraphy data to be included in this study. Participants were 33 to 70 months old and had BMI percentiles scores from 1 to 99. Overall, participants had an average of 8.9 days (i.e., 24-h cycles) of wake and sleep measures (6.8 weekdays and 2.2 weekend days). The average daytime wear time (during wake intervals) was 730.4 min. Sleep outcome measures ranged from 78.7% to 97.2% for sleep efficiency, 0 to 7 days for nap frequency, 7 to 70 for Total Sleep Disturbances score, and 5 to 13 for the Bedtime Resistance score.

The average 24-h movement behavior composition among participants consisted of 47.2% sleep, 22.1% stationary time, 22.8% light physical activity, and 7.9% MVPA. The lowest level of co-dependence was observed between stationary time and MVPA (0.275), and the highest co-dependence was observed between sleep and light physical activity (0.025) (Table S1). Participants without sufficient weekend time that were included in the analysis did not differ in outcome measures from those that did have weekend measures with the exception of nap frequency (i.e., participants without weekend time had slightly greater nap frequency).

### 3.2. Baseline Linear Regression Models

In the baseline compositional linear regression models, the analysis of variance of model parameters indicated that the ILR coordinates of compositional behavior data were significantly associated with sleep efficiency and nap frequency, but not with either of the CSHQ subscales (Table 2).

### 3.3. Time Reallocation Predictions

The differences in predicted outcome estimates between baseline compositions and 30-min pair-wise time reallocations are presented in Table 3. Adding time to sleep while taking away from stationary time or light physical activity was associated with a decrease in sleep efficiency, whereas adding time to stationary time at the expense of MVPA was associated with an increase in sleep efficiency. Adding 30 min to sleep while taking away from stationary time or light physical activity was associated an increase in nap frequency. A decrease in Total Sleep Disturbances score was associated with reallocating time to stationary time from MVPA. A decrease in Bedtime Resistance score was associated with reallocating time to sleep from stationary time or light physical activity.

## 4. Discussion

This study contributes to our limited understanding of the relationships between physical activity and sleep in early childhood by examining outcomes regarding sleep quality and habits with an appropriate analysis (i.e., compositional isotemporal substitution). Overall, the profile (or the combined effect) of an acute measurement of 24-h movement behaviors was associated with sleep efficiency and nap frequency. Given findings from adult studies, when time was reallocated to one behavior from another behavior (while keeping the remaining two behaviors constant) some of the significant effects on the change in predicted sleep outcome estimates were in unexpected directions. For example, increasing MVPA (and adding to stationary time) was linked to a decrease in predicted sleep efficiency (indicating lower sleep quality) and an increase in predicted Total Sleep Disturbances score (indicating more disturbed sleep). However, the majority of time reallocations did not result in significant changes in predicted sleep outcome estimates.

Other observational studies examining relationships between accelerometry-measured physical activity behaviors and sleep efficiency in younger children (i.e., infants) reported mixed findings. One study from Taiwan (*n* = 183) did not observe significant relationships between physical activity measures and sleep efficiency in infants [25], whereas in another analysis of 141 infants from the United Kingdom, physical activity was associated with fewer wake episodes during overnight sleep [24]. However, it should be noted that this relationship did not remain significant when the model adjusted for covariates, and neither of these infant studies adjusted for other physical activity behaviors.

Similarly, studies among older children with the same type of movement behavior measures used in the current study have indicated conflicting reports when examining relationships between physical activity behaviors and sleep efficiency. Ekstedt et al. [54] observed that in preadolescent Swedish children (*n* = 1231), between-participant associations demonstrated that higher MVPA was related to greater sleep interruptions. Conversely, in the same sample, within-participant associations indicated that higher levels of MVPA and lower levels of sedentary time were related to an increase in sleep efficiency the following night. Comparably, in a sample of 275 Finnish 8-year-old children, Pesonen et al. [55] also examined temporal relationships and reported that increases in physical activity during the day were associated with increases in that night’s sleep efficiency. In 7 to 12-year-old girls from low socioeconomic communities in the U.S. (*n* = 55), physical activity was negatively correlated with the number of awakenings at night and sleep fragmentation (i.e., higher physical activity levels related to better sleep quality) [56]. In contrast, some studies reported null associations. For example, no temporal relationships were observed between daytime physical activity and sleep efficiency in a sample of Australian 8 to 11-year-old children (*n* = 65) or among a sample of Slovenian and American 10.5- to 12-year-old children (*n* = 276) [57,58]. Hence, while some studies have observed positive associations between physical activity and sleep efficiency, other reports have indicated negative or null associations.

At least two studies in older children reported a positive association between sedentary time and sleep efficiency, which is somewhat consistent with our results (i.e., an increase in stationary time at the expense of MVPA was associated with higher sleep efficiency). In an international comparison, Chaput et al. [59] reported that sleep efficiency was positively associated with sedentary time (as well as negatively associated with MVPA) in children ages 9 to 11 years old (*n* = 5777), although this association differed between the study sites and countries (i.e., high-income vs low/middle-income) within their study. Correspondingly, using data from the same international study, McNeil et al. [60] found that children with the highest sleep efficiencies had greater sedentary time and lower light physical activity in a sample of 567 10-year-old children. As was suggested by McNeil et al., it is plausible that due to greater movement associated with less sleep efficiency among children during the night (e.g., restlessness and frequent repositioning), children may increase light physical activity (at the expense of sedentary time) as a method to stay awake during the day to compensate for increased daytime sleepiness. Although this is only a hypothesis that has yet to be examined, this could potentially explain to the present study’s positive association between stationary time and sleep efficiency.

In the current study, the overall null associations observed between 24-h behaviors and CSHQ subscales were parallel to other observational studies that examined objectively measured physical activity with the same or similar sleep habits questionnaires. In a sample of Australian toddler-age children (*n* = 173) physical activity measures were not correlated with sleep problems and behaviors as assessed by the Tayside Children’s Sleep Questionnaire. In another toddler study (*n* = 240 U.S. 12 to 32-month-old children), Hager et al. [27] also did not observe any significant associations between MVPA and sleep behaviors as measured with the Brief Infant Sleep Questionnaire. The CSHQ was also used by Greever et al. in 55 preadolescent girls in the U.S. [56], but no significant associations were determined between physical activity level and the total score. Although the resulting decrease in estimated CSHQ subscale scores with time reallocation away from MVPA in the current study was surprising, previous reports in children have not substantiated a positive association between MVPA and sleep disturbances.

Other studies exploring the relationship between physical activity in relation to nap habits are sparse. Hauck et al. [20] examined such associations in 6-month-old infants from the U.S. (*n* = 22) and reported that nap frequency was not correlated with 24-h sleep duration but was negatively associated with an indicator of sedentary behavior (i.e., more sedentary time was associated with less nap frequency). However, associations were from bivariate correlations and were not adjusted for any covariates or behaviors. The positive association between nap frequency and 24-h sleep in our study may seem intuitive (i.e., the more a child naps, the more sleep they will obtain). However, in an previous examination of a subset of the sample used in the current study exploring the associations between nap frequency and nap promotion with other sleep parameters, it was noted that although children that nap more frequently had longer overnight sleep duration, 24-h sleep duration did not differ between nap frequency groups [61].

The current study adds to previous research examining associations between physical activity and sleep duration in early childhood by providing a preliminary insight into other sleep outcomes such as quality and habits. While our findings regarding the CSHQ measures appear to be consistent with other reports, additional studies within a more varied sleep health sample may be warranted given that the majority of our participants had scores that indicated lower sleep problems and disturbances. Furthermore, the analyses used for comparison to this study did not appear to adjust for other 24-h movement behaviors or only occasionally adjusted for MVPA or sleep duration (but with these variables as absolute values), and the samples consisted of infants, toddlers, or preadolescents. Additional studies in preschool age samples that use statistical analysis techniques appropriate for compositional data are warranted to corroborate our findings.

A strength of the present study is that it was one of the first to examine associations of objectively measured 24-h movement behaviors using a compositional isotemporal substitution analysis for such data in an understudied age group in a relatively large sample. Nevertheless, it is also important to note some limitations. First, the inclusion criteria of the parent study may limit our ability to generalize findings to children with sleep disorders. Second, we opted to include participants that did not have at least one day of weekend actigraphy measures because they had such high compliance and valuable data on weekdays. However, the participants that were included had good overall compliance as illustrated by the average wear times. Third, we did not control for screen time in our models, which may act as a confounder in the relationship between 24-h movement behaviors and sleep quality. However, we have separately reported the impact of television on sleep in this population [62]. Finally, as these analyses were cross-sectional, causal relationships cannot be inferred.

## 5. Conclusions

The present study provides preliminary evidence that 24-h movement behavior compositions in early childhood are associated with sleep quality and habits (i.e., napping frequency). However, estimated effects from time reallocations contribute to the overall mixed findings that have been reported in youth samples regarding sedentary behavior, physical activity, and sleep relationships in children. Future studies are warranted to examine the influence of the spectrum of behaviors within the daytime or 24-h period on sleep efficiency and nap habits given the statistical significance observed with these overall compositional models, particularly with longitudinal designs to help explore causal mechanisms underlying these relationships. Such studies should be conducted in samples more diverse in sleep health characteristics and should consider other magnitudes of time reallocation between 24-h movement behaviors.

## Figures and Tables

**Table 1:** Descriptive characteristics of the study sample.

Variables	Mean (SD) or *n* (%)
*Sample Characteristics*	
Age (months)	51.2 (9.6)
Gender (female)	136 (47.2)
BMI percentile (%)	63.5 (28.6)
SES	
Low	52 (18.1)
Middle	71 (24.7)
High	165 (57.3)
Race	
White	178 (65.9)
Black/African American	24 (8.9)
Asian	13 (4.8)
Native Hawaiian/Pacific Isl.	1 (4)
2 or more racial groups	33 (12.2)
Other	21 (7.8)
Hispanic (yes)	74 (26.8)
*24-Hour Movement Behaviors*	
Stationary time (minutes)	318.2
Light physical activity (minutes)	328.4
MVPA (minutes)	113.1
Sleep (minutes)	680.3
*Sleep Outcomes*	
Sleep efficiency (%)	88.3 (8.9)
Nap frequency (#/week)	3.6 (2.0)
Sleep disturbances (score)	43.2 (8.9)
Bedtime resistance (score)	8.4 (1.6)

**Table 2:** Analysis of variance results for sleep outcome baseline linear models.

	Sum Sq	df	F Value	*p*-Value
*Sleep efficiency*				
ILR coordinates	479.71	3	15.5	<0.001
Age	10.0	1	1.0	0.52
Gender	4.2	1	0.4	0.32
BMI percentile	4.9	1	0.5	0.49
*Nap frequency*				
ILR coordinates	52.3	3	4.7	0.003
Age	18.8	1	5.1	0.03
Gender	0.1	1	0.01	0.89
BMI percentile	22.8	1	6.1	0.01
*Total Sleep Disturbances*				
ILR coordinates	483.7	3	2.1	0.10
Age	431.0	1	1.9	0.17
Gender	141.2	1	5.7	0.02
BMI percentile	18.7	1	0.2	0.62
*Bedtime Resistance*				
ILR coordinates	17.5	3	2.4	0.07
Age	12.6	1	5.2	0.02
Gender	0.2	1	0.1	0.79
BMI percentile	0.4	1	0.2	0.70

**Table 3:** Predictive differences in sleep outcomes following reallocation of 30 min between movement behaviors.

	Sleep Efficiency	Nap Frequency	Total Sleep Disturbances	Bedtime Resistance
	Estimate (95% CI)	Estimate (95% CI)	Estimate (95% CI)	Estimate (95% CI)
+ Sleep − ST	**−0.77 (−1.01, −0.52)**	**0.23 (0.08, 0.38)**	−0.05 (−0.75, 0.65)	**−0.13 (−0.26, −0.01)**
+ Sleep − LPA	**−0.78 (−1.14, −0.42)**	**0.35 (0.13, 0.56)**	−0.29 (−1.31, 0.74)	**−0.21 (−0.40, −0.02)**
+ Sleep − MVPA	−0.31 (−0.76, 0.14)	0.20 (−0.07, 0.47)	−1.22 (−2.52, 0.08)	−0.16 (−0.40, 0.07)
+ ST − Sleep	**0.75 (0.51, 0.99)**	**−0.23 (−0.37, −0.09)**	0.07 (−0.61, 0.75)	**0.13 (0.01, 0.25)**
+ ST − LPA	−0.04 (−0.38, 0.29)	0.12 (−0.08, 0.33)	−0.22 (−1.20, 0.75)	−0.08 (−0.26, 0.10)
+ ST − MVPA	**0.42 (0.05, 0.79)**	−0.02 (−0.24, 0.20)	**−1.16 (−2.22, −0.10)**	−0.03 (−0.23, 0.16)
+ LPA − Sleep	**0.76 (0.42, 1.10)**	**−0.33 (−0.54, −0.13)**	0.28 (−0.70, 1.26)	**0.20 (0.02, 0.38)**
+ LPA − ST	−0.03 (−0.35, 0.29)	−0.10 (−0.29, 0.10)	0.22 (−0.72, 1.26)	0.06 (−0.11, 0.24)
+ LPA − MVPA	0.43 (−0.13, 0.99)	−0.13 (−0.46, 0.21)	−0.95 (−2.57, 0.68)	0.03 (−0.26, 0.33)
+ MVPA − Sleep	0.34 (−0.02, 0.70)	−0.19 (−0.41, 0.02)	0.98 (−0.07, 2.02)	0.15 (−0.04, 0.34)
+ MVPA − ST	**−0.45 (−0.73, −0.16)**	0.04 (−0.13, 0.21)	**0.92 (0.10, 1.73)**	0.01 (−0.14, 0.16)
+ MVPA − LPA	−0.45 (−0.95, 0.04)	0.16 (−0.14, 0.46)	0.68 (−0.75, 2.12)	−0.06 (−0.32, 0.20)

Key: + = a 30-min addition of behavior; − = a 30-min reduction of behavior; CI = confidence interval; ST = stationary time; LPA = light physical activity; MVPA = moderate to vigorous physical activity; Bold text = statistically significance (*p* < 0.01).

## Supplementary Materials

The following are available online at https://www.mdpi.com/1660-4601/17/19/7148/s1, Table S1: Pair-wise log-ratio variation matrix of the 24-h movement behaviors.
