# Supplementary material for: Cross-Sectional Associations of 24-Hour Sedentary Time, Physical Activity, and Sleep Duration Compositions with Sleep Quality and Habits in Preschoolers
Source: Int J Environ Res Public Health. 2020 Sep 29;17(19):7148. doi: 10.3390/ijerph17197148 (PMC7579350; doi:10.3390/ijerph17197148)
Supplement: Supplementary file 1 [file ijerph-17-07148-s001.pdf]

Table S1. Pair-wise log-ratio variation matrix of the 24-hour movement behaviors.

	**Sleep**	**ST**	**LPA**	**MVPA**
Sleep	0	0.069	0.025	0.147
ST	0.069	0	0.085	0.275
LPA	0.025	0.085	0	0.122
MVPA	0.147	0.275	0.122	0

Key: ST = stationary time; LPA = light physical activity, MVPA = moderate-to-vigorous physical activity.
